# Efficacy of ^18^F-Fluorodeoxyglucose positron emission tomography/computed tomography for detecting renal cell carcinoma in patients with end-stage renal disease

**DOI:** 10.1007/s11604-024-01593-5

**Published:** 2024-05-25

**Authors:** Hiromi Hirasawa, Ayako Taketomi-Takahashi, Natsumi Katsumata, Tetsuya Higuchi, Yoshitaka Sekine, Kazuhiro Suzuki, Yoriaki Kaneko, Keiju Hiromura, Yasuhiro Fukushima, Yoshito Tsushima

**Affiliations:** 1 Department of Diagnostic Radiology and Nuclear Medicine, Gunma University Graduate School of Medicine, 3-39-22 Showa, Maebashi, Gunma 371-8511 Japan; 2 Department of Urology, Gunma University Graduate School of Medicine, Maebashi, Japan; 3 Department of Nephrology and Rheumatology, Gunma University Graduate School of Medicine, Maebashi, Japan; 4 Department of Applied Medical Imaging, Gunma University Graduate School of Medicine, Maebashi, Japan

**Keywords:** Renal cell carcinoma, End-stage renal disease, FDG-PET/CT, Screening

## Abstract

**Purpose:**

Dialysis patients are at an increased risk of developing renal cell carcinoma (RCC); however, differentiating between RCC and benign cysts can sometimes be difficult using modalities, such as computed tomography (CT) and ultrasonography. ^18^F-Fluorodeoxyglucose positron emission tomography (FDG-PET)/CT efficiently detects malignant tumors; however, physiological accumulation of FDG in the kidney limits its efficacy in detecting renal tumors. However, in patients with severely impaired renal function, the renal accumulation of FDG is decreased, possibly improving the detection of renal malignancies in this patient population. This study evaluated the usefulness of FDG-PET/CT as a screening tool for detecting RCC in patients with end-stage renal disease.

**Materials and methods:**

This prospective study recruited 150 participants from 2012 to 2016 who were on dialysis or underwent renal transplantation and were on dialysis until transplantation. FDG-PET/CT was performed to screen for RCC. Three radiologists independently evaluated the images. No protocol was defined for the additional management of positive examinations, leaving decisions to the discretion of each participant. Negative examinations were observed until the end of 2019.

**Results:**

In total, 150 participants (mean age, 58 ± 13 years; 105 men) underwent FDG-PET/CT. Twenty patients (13.4%) were diagnosed as positive. Fifteen patients underwent additional examinations and/or procedures, and RCC was found in seven patients. Of the four patients who underwent surgical resection, the pathological results were clear cell RCC in one, papillary RCC in one, and acquired cystic disease-associated RCC in two. Two participants were diagnosed with RCC on bone biopsy, and one was diagnosed on dynamic CT but opted for observation. The sensitivity, specificity, and negative predictive value were 100%, 93.9%, and 100%, respectively.

**Conclusion:**

FDG-PET/CT was useful for detecting RCC in patients with end-stage renal disease. Our findings show the potential use of FDG-PET/CT as a screening tool for RCC in this patient population.

## Introduction

The high incidence of malignancies in hemodialysis patients is well known. A recent report stated that when 639 dialysis patients were followed for a median of 5.61 years, 58 developed malignancies, which is 1494 cases per 10^5^ person-years [1]. Gastrointestinal malignancies were the most common (32.8%), followed by urological malignancies (19.0%).

Among renal transplant patients, an assessment of 260 kidneys removed at the time of transplantation found renal cell carcinoma (RCC) in 4.2% [2]. When the cause of renal failure is autosomal-dominant polycystic kidney disease (ADPKD), the rate of RCC doubles, with seven of 84 kidneys found to have RCC [3], and lesions were reported to be frequently bilateral or multiple [3, 4]. Improved dialysis technology has led to longer life spans, and early detection of malignant neoplasms is likely to become an important prognostic factor.

Kidneys with end-stage renal disease frequently have benign cysts. The frequency of acquired cystic kidney disease (ACKD) varies among reports, with one report stating an incidence of 44% when patients have been on dialysis for < 3 years, 79% for patients on dialysis for > 3 years, and 90% for patients on dialysis for ≥ 10 years [5, 6]. Detecting RCC in kidneys with multiple cysts using computed tomography (CT), magnetic resonance imaging (MRI), and ultrasonography (US) is highly cumbersome. This is not only because of the large number of cysts in these patients’ kidneys but also because of the difficulty in differentiating benign cysts complicated by hemorrhage or infection (an unfortunately common phenomenon in ADPKD and ACKD) from RCC. A screening test for dialysis patients to detect RCC in its early stages would be highly beneficial.

^18^F-Fluorodeoxyglucose positron emission tomography (FDG-PET)/CT is widely used in the detection, staging, and evaluation of therapeutic efficacy in malignant tumors. Although it is considered useful in detecting metastatic lesions [7, 8], the efficacy of FDG-PET/CT in detecting and evaluating primary urological tumors is limited by the physiological renal uptake and excretion of FDG. FDG accumulation in the kidneys and excretion into the urinary tract is predictably decreased in dialysis patients, leaving potential for tumors in the kidneys and urinary tract to accumulate sufficient FDG for detection. In fact, some reports have stated that FDG-PET is useful for diagnosing RCC in patients on dialysis [9, 10].

Therefore, this study evaluated the efficacy of FDG-PET/CT as a screening tool for diagnosing RCC in dialysis patients.

## Materials and methods

### Participant selection

For this prospective study, we recruited 150 dialysis patients from 2012 to 2016 through leaflets placed in area hospitals with dialysis facilities. The conditions for participation were as follows: 1) 18 years of age or older and currently undergoing dialysis or having undergone renal transplantation but had undergone dialysis until that time, 2) not pregnant or suspected of being pregnant, and 3) able to come to our facility to undergo FDG-PET/CT. We did not specify the duration of dialysis or the cause of kidney disease and excluded those who had already been diagnosed with RCC by previous imaging examinations.

Patient data, such as age, sex, cause of kidney disease leading to dialysis, and duration of dialysis, were obtained by reviewing patient records.

### FDG-PET/CT examination

FDG-PET/CT examinations were performed from 2012 to 2016 using either Discovery ST 16 (GE Healthcare Japan, Tokyo, Japan) or Biograph 16 (Siemens Japan, Tokyo, Japan). FDG was synthesized using an on-site cyclotron unit (Cypris HM-18, Sumitomo Heavy Industry Co. Ltd., Tokyo, Japan). The FDG dose was 5 MBq/kg, the standard for clinical examinations at our institution. Imaging was performed after a rest period of 60 min and to include areas from the top of the head to the inferior margin of the pelvis. The participants were instructed to fast for at least 6 h before FDG injection.

### FDG-PET/CT evaluation

Three board-certified radiologists, who interpreted clinical nuclear medicine studies daily, independently evaluated the FDG-PET/CT images. They also reviewed coronal maximum intensity projection (MIP) and axial images. Axial images were fused with CT images to confirm whether or not abnormal uptake was due to a renal tumor. Images were evaluated visually for uptake that was suspicious for RCC, and semiquantitative evaluation was performed on uptake that was considered suspicious. The decision of whether an uptake was suspected of being RCC was left to the radiologist reviewing the images, as it was difficult to set a cutoff value with any reliability. Semiquantitative evaluation consisted of the SUV_max_ of the uptake in question [SUV_max_ (tumor)], the mean uptake of renal parenchyma outside the tumor [SUV_mean_ (renal parenchyma)], and the ratio of these values (SUV ratio), i.e., SUV_max_ (tumor)/SUV_mean_ (renal parenchyma) = SUV ratio.

If one or more radiologists (of three) made a diagnosis of RCC, the study was considered positive. When two or more radiologists concluded that the study was positive, the SUV ratio derived by each radiologist were averaged.

### Patient notification of the results

The results of the FDG-PET/CT examination were reported to each participant and the referring physician. The decision to undergo additional evaluation or therapy was made by each participant.

### Final diagnosis

RCC on final diagnosis was defined as masses in patients diagnosed with RCC on FDG-PET/CT for which one or more of the following applied: 1) enhanced CT and/or non-contrast-enhanced MRI findings of the FDG-accumulating lesion were consistent with RCC (lesions with an obvious solid component, equivalent to Bosniak grade III or higher), 2) pathological diagnosis of the surgical specimen was RCC, 3) pathological diagnosis of a needle biopsy specimen from the lesion in question was RCC, and 4) a metastatic tumor was pathologically diagnosed as carcinoma, and FDG-PET/CT did not identify any potentially malignant lesions other than the mass identified in the kidney.

When the participants were suspected of having RCC on FDG-PET/CT but additional examinations ruled out that possibility, they were followed closely, and subsequent CT and/or MRI examinations were performed.

### Clinical follow-up

The subsequent management of participants not diagnosed with RCC was decided by the referring physician after discussion with the patient, and there was no study-determined protocol for this management. The observation period was set to be until the end of 2019, and the survival of the participant and diagnosis of RCC (or lack thereof) were confirmed. Participants who were not diagnosed with RCC on FDG-PET/CT and did not receive a diagnosis of RCC during the subsequent follow-up period until the end of 2019 were considered true negatives. The follow-up period was 45 ± 15 months (range, 11–73 months).

### Data analysis

The prevalence of RCC among individuals with end-stage renal disease was assumed to be 4.2%, based on previous studies. The number of cases necessary to prove this was calculated at a confidence level of 99% with an interval of 0.10, which was 120. Considering the possibility of dropouts, the required number of cases was set at 150.

Participants diagnosed as positive on FDG-PET/CT were divided into those with a final diagnosis of RCC and those for whom RCC was ruled out. FDG-PET/CT findings were compared. We evaluated the accuracy, sensitivity, specificity, and false-positive and false-negative results of FDG-PET/CT for diagnosing RCC. Data are shown as descriptive statistics and were analyzed by performing the Brunner–Munzel test. For statistical analysis, we used EZR [11]. EZR is a statistical analysis software that expands the functions of R and R Commander.

### Ethical review

This study was approved by our Institutional Review Board. The participants were presented with the purpose, risks, and benefits of this study in oral and written formats and provided written informed consent. The participants did not incur the cost of examinations for this study and did not receive financial compensation for participation.

## Results

### Participants

**Fig. 1:**
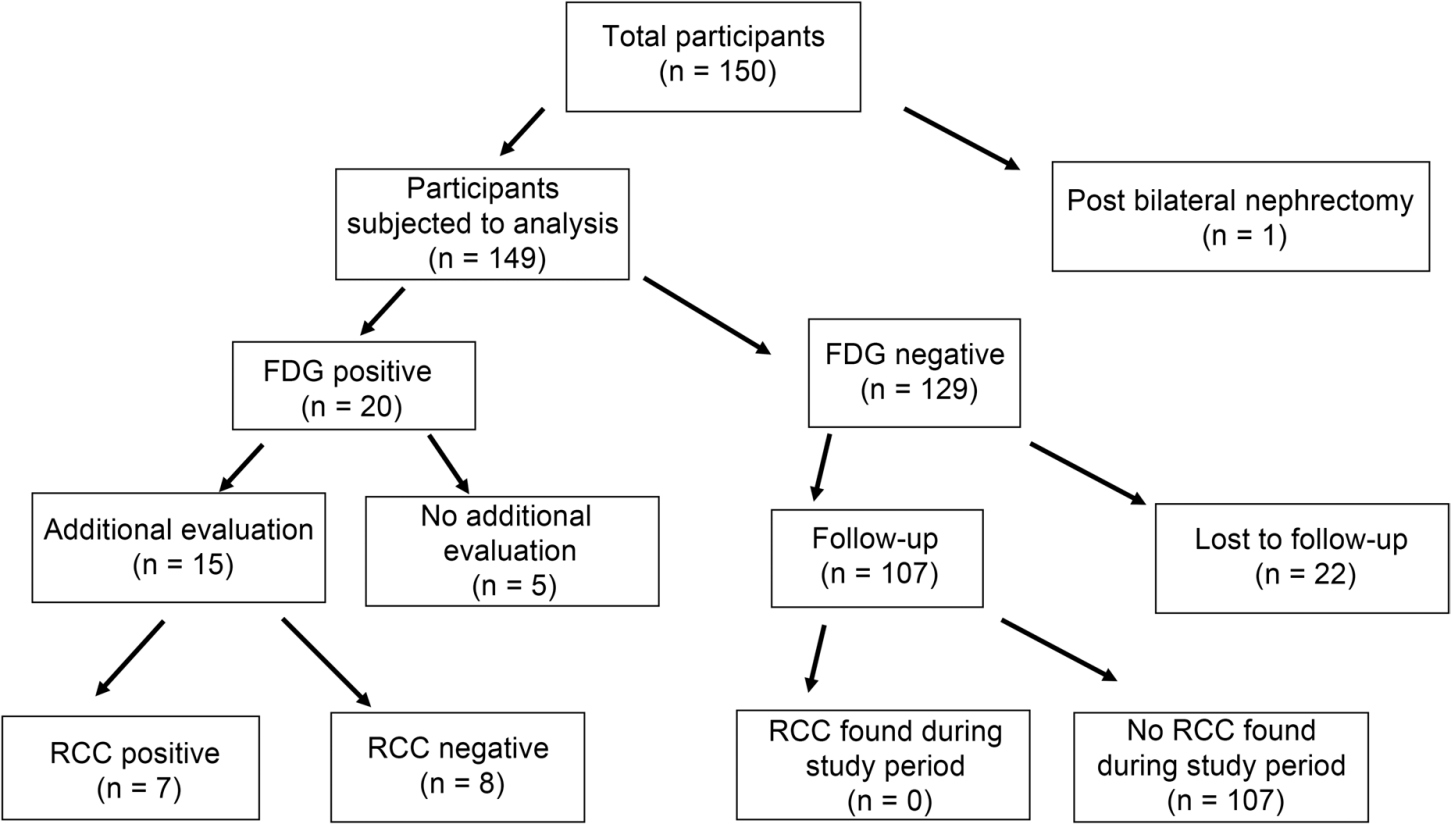
Flow chart of the participants. We recruited 150 dialysis patients from area hospitals to undergo ^18^F-Fluorodeoxyglucose positron emission tomography (FDG-PET)/CT and analyzed 149 patients. Fifteen FDG-positive participants underwent additional evaluation, and seven were RCC-positive. Of the 129 FDG-negative participants, no RCC was found in 107; however, 22 participants were lost to follow-up

In this study, 150 participants (105 men and 45 women), with a mean age of 58 ± 13 years (range, 28–88 years), were enrolled. One patient underwent bilateral nephrectomy before FDG-PET/CT examination and was excluded from the study. Figure 1 shows a flow chart of the study participants showing the number of participants, FDG-PET/ CT results, final diagnosis, and clinical course.

The cause of renal failure requiring dialysis and the duration of dialysis for the remaining 149 participants are shown in Table 1.

**Table 1:** Causes of kidney disease in participants

Cause of kidney disease	No. of patients (M:W)	Age (y.o.) (range)	Duration of dialysis (months) (range)
Chronic nephritis	61 (45:16)	60.1 ± 13.9 (28–86)	139.2 ± 102.5 (1–408)
Diabetic nephropathy	25 (21:4)	58.3 ± 11.9 (36–75)	67.3 ± 51.1 (7–216)^a^
ADPKD	24 (14:10)	61.6 ± 9.0 (46–79)	109.0 ± 73.3 (10–300)
IgA nephropathy	10 (6:4)	48.6 ± 9.44 (38–64)	71.7 ± 62.7 (9–195)
Gout	2 (1:1)	62, 71	13, 164
Hypertensive nephrosclerosis	4 (3:1)	28, 30, 45, 72	18, 19, 112, 240
Acute nephritis	2 (1:1)	39, 50	9, 51
Gestational hypertension	2 (0:2)	59, 69	102, 396
VUR	2 (1:1)	34, 35	3, 67
Other^b^	4 (3:1)	47, 49, 50, 59	23, 67, 96, 204
Unknown	13 (9:4)	61.3 ± 14.3 (38–88)	96.8 ± 86.1 (4–328)^a^

*ADPKD* autosomal dominant polycystic kidney disease; *VUR* vesicoureteral reflux

^a^Exact duration unknown in one participant

^b^Other causes were multiple myeloma, pyelonephritis, bilateral hydronephrosis, and nail–patella syndrome

The most common cause of renal failure was chronic nephritis (61 patients; 40.9%), followed by diabetic nephropathy (25 patients; 16.8%) and ADPKD (24 patients; 16%). The duration of dialysis was unclear in two participants, and among the 147 participants in which it could be confirmed, the duration of dialysis was 111 ± 92 months (median, 96 months; range 1−396 months).

### Positive for RCC on FDG-PET/CT and final diagnosis

**Fig. 2:**
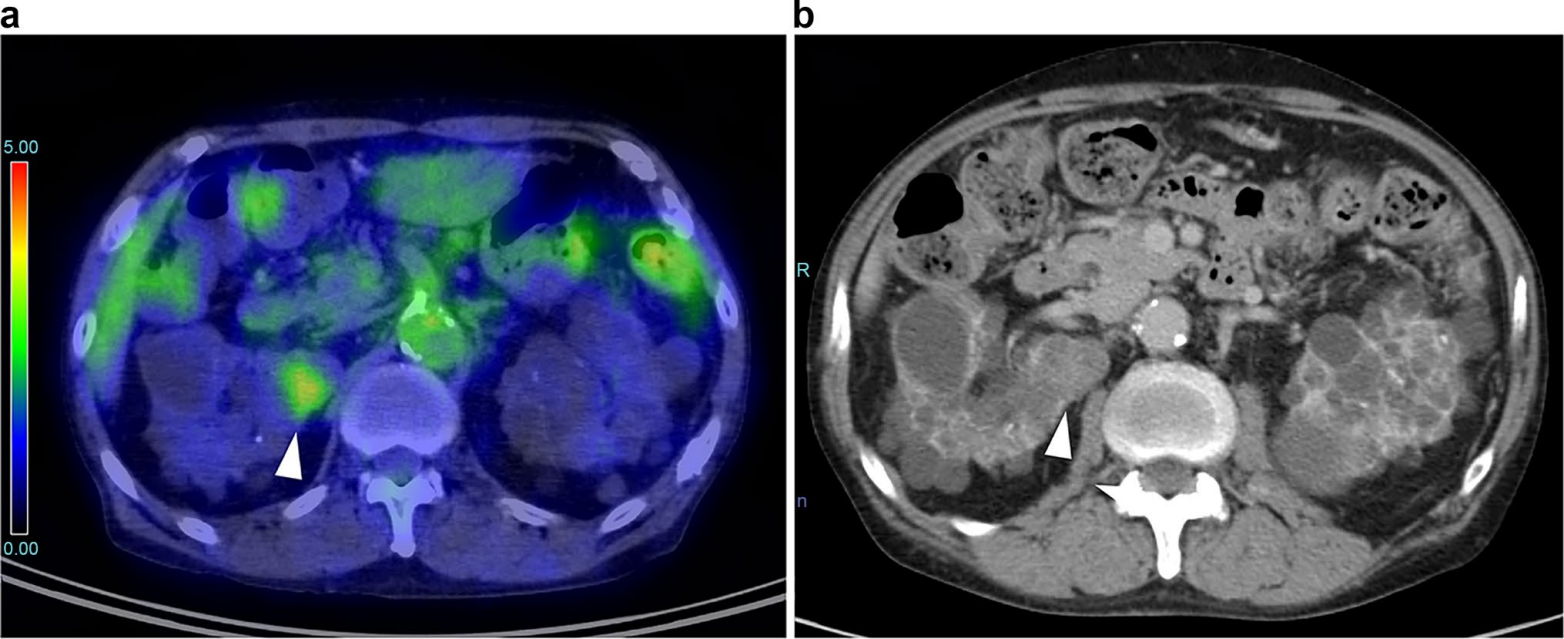
68-year-old man with surgically-proven ACD-RCC. **a** Axial FDG-PET/CT shows a focal accumulation in the right kidney with an SUV_max_ of 3.3 (arrow). **b** Contrast-enhanced CT shows a right renal mass, consistent with the accumulation showing weak enhancement (arrow)

Twenty tumors in 20 participants (age, 60.7 ± 10.2 years; range, 42–84 years) were diagnosed as positive. A typical positive FDG-PET/CT examination is shown in Fig. 2. Among these 20 participants, 15 underwent additional examinations, and seven received a final diagnosis of RCC (4.7%) (Table 2). In the four participants who underwent nephrectomy, the pathological diagnoses were ACD-associated renal cell carcinoma (ACD-RCC) in two, clear cell RCC in one, and papillary RCC in one. In two participants, metastatic lesions were biopsied and found to be consistent with RCC on pathology. In the remaining participant, a hypervascular tumor consistent with RCC was identified on contrast-enhanced dynamic CT; however, after discussion with the participant, additional therapy was not performed, and the participant was subsequently followed without active intervention.

**Table 2:** Participants with definitive diagnosis of RCC

Patient No	Age (years)/sex	Diagnostic method	Pathological diagnosis	Size of renal tumor (cm)	Duration of dialysis (months)
1	68/M	Nephrectomy	ACD-RCC	3.8 × 2.4	300
2	69/M	Needle biopsy of bone metastasis	Consistent with metastasis of RCC	7.7 × 5.6	408
3	69/M	Nephrectomy	Papillary RCC	3.2 × 3.0	171
4	46/M	Nephrectomy	Clear cell RCC	2.5 × 2.8	120
5	67/M	Needle biopsy of bone metastasis	Consistent with metastasis of RCC	3.5 × 3.3	192
6	84/M	Dynamic CT	N/A	3.0 × 2.4	185
7	56/M	Nephrectomy	ACD-RCC	6.1 × 5.2	360

*ACD-RCC* acquired cystic disease associated renal cell carcinoma

The cause of renal failure was chronic nephritis for participants 2, 5, 6, and 7, ADPKD for participants 1 and 4, and unknown for participant 3

**Fig. 3:**
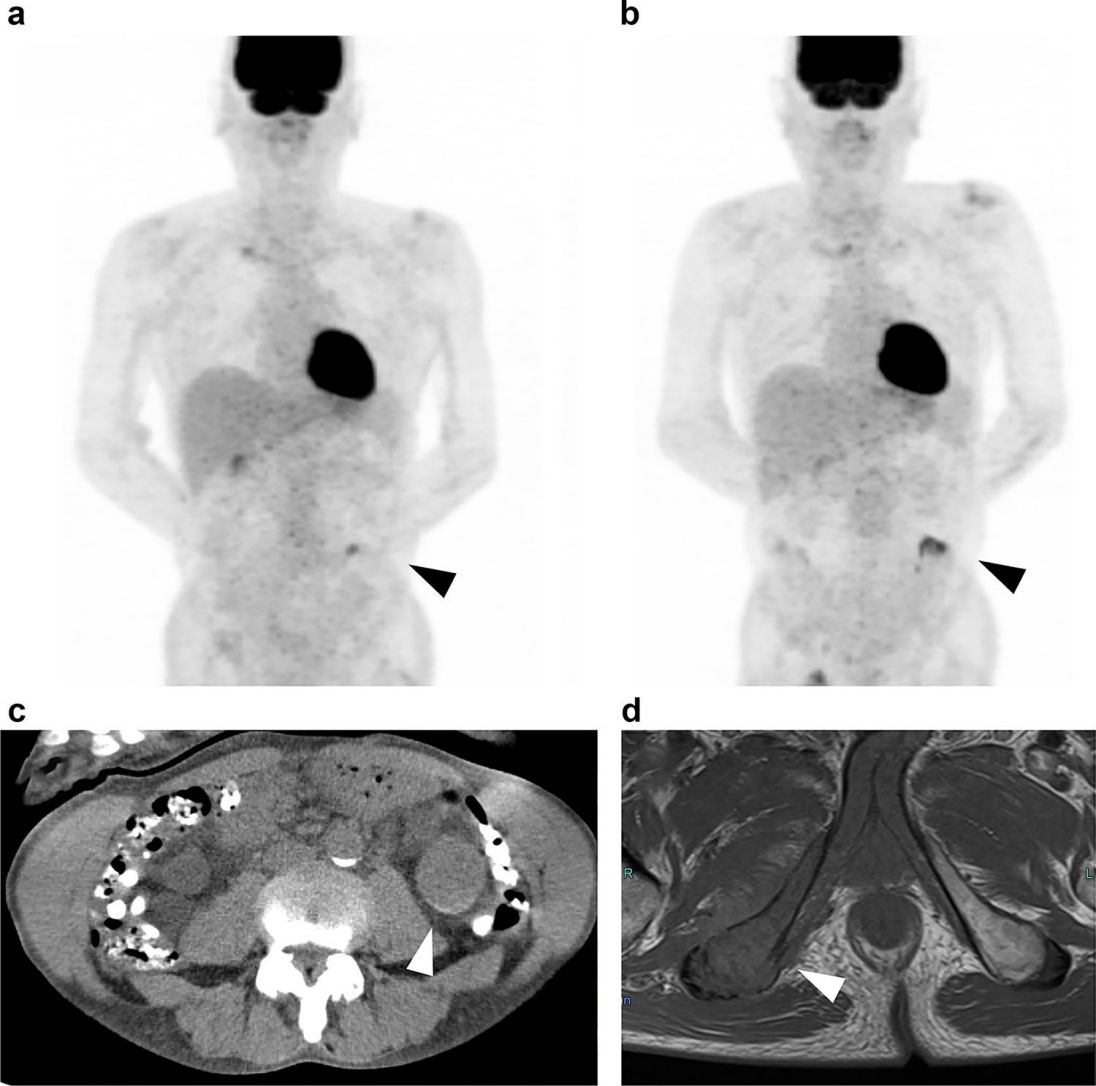
67-year-old man with pathologically proven RCC metastasis in the right ischial bone. **a** FDG-PET: Maximum intensity projection (MIP) image of initial FDG-PET/CT study shows a focal accumulation with an SUV_max_ of 4.0 consistent with the inferior pole of the left kidney (arrow). **b** FDG-PET (MIP image) 27 months after the initial investigation shows the accumulation consistent with the left kidney has increased in size, with an SUV_max_ of 5.0 (arrow). **c** Non-contrast-enhanced CT at the time of study participation had no findings suggesting malignancy. **d** Axial T1-weighted image of the pelvis 27 months after study participation depicted a hypointense lesion in the right ischial bone (arrow). This lesion was biopsied, and the pathological diagnosis was found to be consistent with RCC metastasis

One of the two participants diagnosed as RCC based on biopsy of metastatic lesions, a 67-year-old man, had an FDG-PET/CT positive for RCC but was diagnosed as not having RCC on nonenhanced MRI. Two years and 3 months later, a tumor was found in the patient’s ischial bone. The tumor was biopsied and pathologically diagnosed as metastatic carcinoma. Because no other suspicious lesions were found, the lesion positive for RCC on FDG-PET/CT was considered to be the primary tumor. The participant died of this disease despite subsequent treatment (Fig. 3).

**Fig. 4:**
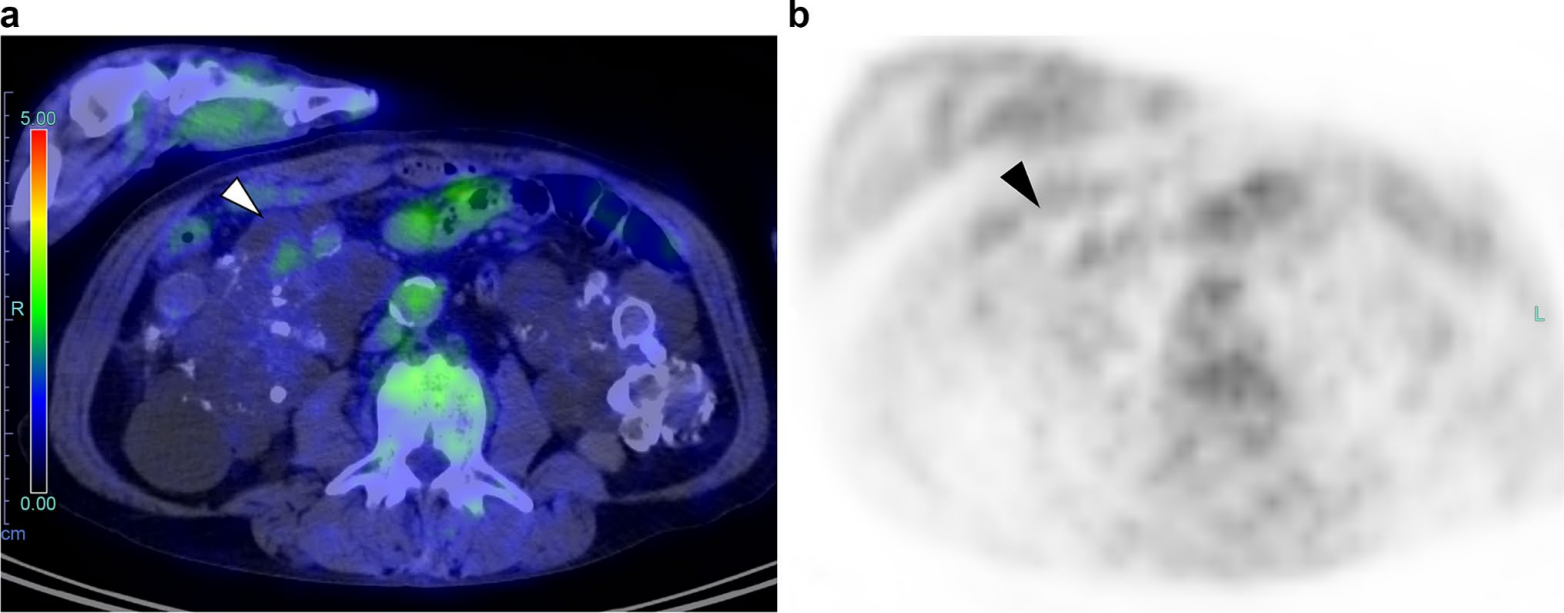
False-positive examination of a 62-year-old man FDG-PET/CT axial (**a**) and FDG-PET axial (**b**) images show FDG uptake with SUV_max_ of 2.0 in the right kidney (arrows). Additional examinations (non-contrast-enhanced MRI and ultrasonography) were performed; however, no tumor was identified. As of 4 years and 11 months of observation, no tumor has been identified. The cause of kidney disease was ADPKD

Eight of the fifteen participants were not diagnosed with RCC on additional examinations or nephrectomy (four patients diagnosed on contrast-enhanced CT, two on non-contrast-enhanced MRI, one by biopsy, and one by surgical resection) (Fig. 4). Of the 20 participants who were positive on FDG-PET/CT, five chose not to undergo additional examinations or therapy and opted for clinical observation.

The agreement rate of the radiologists was 92.6% (138/149).

### Size and SUV_max_ ratios of detected tumors

The greatest diameter on axial CT or MRI images of the 19 positive lesions was 2.9 ± 1.9 cm (range, 0.8–7.7 cm). One positive lesion was small, had poorly defined margins, and could not be measured. This tumor was not included in the statistical analysis. The seven tumors with a final diagnosis of RCC had a mean diameter of 4.3 ± 1.7 cm (range, 2.8–7.7 cm). Of the eight lesions not considered to be RCC, the diameters of seven could be measured and were 2.1 ± 1.3 cm (range, 0.8–4.0 cm). The five lesions that did not undergo additional examinations had a mean diameter of 2.2 ± 1.7 cm (range, 1.0–5.5 cm).

Of the 20 lesions, the mean SUV ratio was 2.9 ± 1.3 (range, 1.3–6.0); of the RCCs, the mean SUV ratio was 3.6 ± 0.5 (range, 2.5–5.3), and that of the lesions not considered to be RCC was 2.7 ± 1.2 (range, 1.8–6.0). No significant difference in size (*p* = 0.0502) was observed between RCCs and lesions not considered to be RCC, but there was a significant difference in the SUV ratios (*p* = 0.0302).

### Clinical course of participants negative for RCC on FDG-PET/CT

The 129 participants who were negative for RCC on FDG-PET/CT were followed clinically; however, 22 were lost to follow-up (12 died during the follow-up period, and 10 were lost to follow-up due to relocation and/or transfer to different institutions). In total, 107 participants were confirmed to be alive at the end of 2019, none of whom had been diagnosed with RCC. The observation period was 43.7 ± 15.9 months (range, 5–73 months). These 107 participants and the eight who were positive for RCC on FDG-PET/CT but were found not to have RCC on additional evaluation (*n* = 115 participants) were considered true negatives for RCC. Among the eight participants positive for RCC on FDG-PET/ CT, one had RCC on CT 4 years and 6 months after the initial FDG-PET/ CT. However, as this RCC was contralateral to the uptake found on the initial FDG-PET/ CT, we decided that this was a new RCC that developed during the follow-up period; therefore, the participant was counted as a true negative.

The cause of death was aortic dissection in one participant. The causes of death in the remaining 11 patients could not be confirmed.

### Sensitivity, specificity, positive predictive value, and negative predictive value of FDG-PET/CT

On exclusion of the six participants in whom additional evaluation was not performed (either due to participant preference or physician discretion) and the 22 participants lost to follow-up, the sensitivity was 100% (7/7), specificity was 93.0% (107/115), positive predictive value was 46.7% (7/15), and negative predictive value was 100% (107/107).

## Discussion

In this cross-sectional study of dialysis patients, we diagnosed RCC in 7 (4.7%) of the 149 participants included in this study using FDG-PET/CT. This value is consistent with previous reports on the prevalence of RCC among dialysis patients (4.2%) [2]. FDG-PET/CT also showed high sensitivity (100%) and specificity (93%) for the diagnosis of RCC. The high negative predictive value (100%) suggested that this examination is sufficient as a screening tool. There was a significant difference in SUV ratios between the RCC-positive and the RCC-negative groups, but there was a large variance among values; therefore, the clinical utility of this finding is limited.

RCC histology in dialysis patients has been reported to have a distinct tendency. In patients on dialysis for < 10 years found to have RCC, clear cell RCCs have been reported to be the most common, just as they are in non-dialysis patients. In this patient population, 17–18% of RCCs were reported to be ACD-RCCs, although in patients on dialysis for ≥ 10 years, a much higher percentage (46%) of RCCs were ACD-RCCs [5, 12, 13]. Papillary RCCs have also been reported to increase with the duration of dialysis [14]. Papillary RCC is hypovascular on contrast-enhanced CT [15], and ACD-RCC shows equivocal or mild enhancement on imaging [13], making their detection among multiple (frequently hemorrhagic) cysts difficult. These characteristics make FDG-PET a potentially useful screening tool for RCC in patients on dialysis for ≥ 10 years.

Magnetic resonance diffusion-weighted imaging (MRI-DWI) may be more widely available than FDG-PET/CT, making it potentially useful for RCC screening. However, infected and hemorrhagic cysts also have restricted diffusion, potentially contributing to a higher false-positive rate. Studies have examined on the efficacy of choline PET for diagnosing RCC, stating its clearer uptake than FDG-PET [16]. The false-positive rate of choline PET may be lower than that of FDG-PET (because it does not accumulate in infection); however, it is less accessible than FDG-PET/CT, making it a less appropriate screening tool.

Even with the decreased FDG uptake in patients on dialysis, residual normal renal parenchyma can complicate the interpretation of low uptake, making it challenging to distinguish between cancerous and physiologic uptake. Therefore, these images should be interpreted by experienced nuclear medicine physicians. The development of a tracer to replace FDG in tumor detection has long been a focus of researchers. Recent advancements include amino acid tracers that reflect the vivid metabolism of cancer cells and tracers targeting ligands specific to cancer cells. For instance, prostate specific membrane antigen (PMSA), used specifically to diagnose prostate cancer, has been reported to accumulate in RCC, showing an affinity for clear cell cancer in particular [17]. However, clear cell cancer is easily identifiable on CT, limiting the utility if PSMA in this context. Papillary carcinoma and ACD-RCC, which enhance poorly on CT, would benefit from a tracer that accumulates in these cancers for early diagnosis. Unfortunately, PMSA reportedly does not accumulate in papillary carcinoma, and its uptake (or lack thereof) in ADC-RCC has not been adequately studied. This situation limits PMSA’s utility in patients on dialysis, in whom these cancers are more prevalent [17]. Another tracer whose utility in diagnosing RCC has been examined is [68 Ga]Ga-fibroblast activation protein inhibitor-04([68 Ga]Ga-FAPI-04). Compared to FDG-PET, [68 Ga]-FAPI-04 has limited physiologic accumulation in the gastrointestinal tract and kidneys and less excretion into urine [18]. Civan et al. [19] reported primary tumors, local recurrence, and most metastatic lesions of RCC showed a higher SUV_max_ for FAPI compared to FDG, but that lymph node metastases of RCC showed a higher SUV_max_ for FDG compared to FAPI. Given the severe decrease in renal function (and FDG excretion) in patients on dialysis, FDG is potentially useful in this limited setting. Conversely, decreased excretion of FAPI into urine might make the detection of RCC even easier. Further studies comparing the potential of FAPI-PET for diagnosing RCC in patients on dialysis with that of FDG-PET are warranted.

In this study, the negative predictive value of FDG-PET/CT for RCC was considered sufficiently high (100%) for screening examination. However, the positive predictive value was low (46.6%) and the false-positive rate was high. Of the eight false-positive participants in our study, one had a renal mass and uptake in the renal vein and paraaortic, mediastinal, and bilateral supraclavicular lymph nodes. The renal mass was biopsied under CT guidance; however, we could not diagnose a malignant tumor, and all lesions spontaneously regressed on clinical follow-up. Although rare, there have been reports of spontaneously regressing RCC [20, 21], and this may have been such a case. Biopsy may have upregulated damage to tumor vascularity [22]. Another case was suspicious for RCC and surgically resected; however, pathology showed atypical epithelium. This lesion was considered to be a precancerous lesion.

FDG accumulation reflects an increase in glucose metabolism, and FDG accumulates not only in tumors but also in infections. The false positives in our study may have also been because of FDG accumulation in infectious lesions. In a report on renal infection of ADPKD or multiple renal cysts diagnosed by FDG-PET, nine of 10 patients exhibited symptoms [23]. None of the false-positive participants in our study complained of fever or abdominal pain; however, we did not perform additional examinations, such as laboratory studies, and the possibility of mild infections remains. These false positives may have been prevented by more detailed patient interviews and additional laboratory studies.

There may be another mechanism for false positives, although only in cases involving ADPKD. In ADPKD, the normal parenchyma remaining between each of the innumerable cysts will have FDG uptake, potentially increasing false positives. In fact, of the six false-positive participants in this study, three had ADPKD. Furthermore, 16% (24/149) of the participants had ADPKD, and these participants may be more likely to show false-positive results than participants with chronic kidney disease of other causes.

Patients on dialysis have minimal FDG excretion into urine, potentially increasing radiation dose. In this study, the protocol standard of our institution was used, and the issue of radiation dose was not explored. Further studies exploring radiation dose optimization would be beneficial to reduce unnecessary risk.

This study had several limitations. First, selection bias was unavoidable. Participants were recruited by displaying leaflets in area hospitals; however, dialysis physicians may have encouraged participation in patients they thought to be at a higher risk of RCC, inadvertently recruiting a higher number of participants with RCC than actually found in patients on dialysis. We did not have a protocol for additional evaluation or clinical follow-up after the FDG-PET/CT examination, leaving these to the discretion of the referring physician and the patient. This situation led to the second and third limitations. The second limitation is that we cannot ascertain if the follow-up period in this study was sufficient. This study was prospective, and recruiting 150 participants took time, leading to a limited observation period in some participants. Third, proving true negatives is challenging. All participants lost to follow-up were in the FDG-PET negative group, raising the possibility that false negatives were among these participants. Among the 107 FDG-negative participants and the eight false-positive participants, only one RCC was detected. This finding is inconsistent with the known RCC prevalence of 4.2% among patients on dialysis. In clinical practice, US is likely the primary screening tool for RCC. Renal US of patients on dialysis may be challenging due to multiple cysts and calcifications, potentially resulting in the oversight of some RCCs. This study intentionally avoided a rigid follow-up protocol, leaving subsequent clinical decisions, including modality and frequency of imaging for follow-up, to referring physicians. Depending on availability, some hospitals performed annual ultrasound, while others performed contrast-enhanced CT or non-enhanced MRI. The flexible protocol allowed participants to be followed-up at the hospitals where they were recruited, making it easier for them to participate in the study. We hope for future large-scale, long-term studies with robust protocols which include specific timelines and imaging modality (or modalities) for follow-up.

In conclusion, we conducted a cross-sectional study of FDG-PET/CT on dialysis patients and found RCC in 4.7% of the participants. This prevalence was obviously higher than that in the general population. This method exploits the lack of physiological FDG accumulation in the urinary tract, and this study showed the potential of this modality in detecting RCC in this patient population. Additional large-scale studies are required to prove its efficacy as a screening tool.
